# Complete recovery after fulminant myocarditis in a patient with COVID-19

**DOI:** 10.12669/pjms.40.4.8685

**Published:** 2024

**Authors:** Vasiliki Kitsou, Torbjørn Lunde, Atle Solholm, Bjørn Blomberg, Sahrai Saeed

**Affiliations:** 1 Vasiliki Kitsou, Department of Heart Disease, Emergency Care Clinic, Haukeland University Hospital, Bergen, Norway; 2 Torbjørn Lunde, Department of Heart Disease, Haukeland University Hospital, Bergen, Norway; 3 Atle Solholm, Department of Heart Disease, Haukeland University Hospital, Bergen, Norway; 4 Bjørn Blomberg, Department of Clinical Science, The Medical Faculty, University of Bergen, Bergen, Norway. National Advisory Unit for Tropical Infectious Diseases, Department of Medicine, Haukeland University Hospital, Bergen, Norway; 5 Sahrai Saeed, Department of Heart Disease, Haukeland University Hospital, Bergen, Norway

**Keywords:** Cardiovascular complications, COVID-19, Echocardiography, Myocarditis

## Abstract

The clinical spectrum of Coronavirus disease 2019 (COVID-19) varies from asymptomatic infection to severe disease with multiorgan dysfunction. Cardiovascular involvement is common and in rare cases can lead to serious complications, such as fulminant myocarditis. The clinical course of COVID-19 myocarditis varies from complete recovery to death in rare cases. The pathophysiology of COVID-19-related myocarditis is still unclear but is believed to involve direct viral injury and cardiac damage due to the host’s immune response. Guidelines on the management of COVID-19-related myocarditis are yet to be established. We present here the case of a male patient in his early fifties admitted with life-threatening myocarditis in the course of COVID-19 infection who was successfully treated and recovered without any sequelae.

## INTRODUCTION

The clinical spectrum of Coronavirus disease 2019 (COVID-19) caused by severe acute respiratory syndrome Coronavirus-2 (SARS-CoV-2) varies from asymptomatic infection to multiorgan involvement of varying degrees of severity, and with potentially fatal outcome in some high-risk patients.^1^ Although the respiratory system is the main entry for the coronavirus, other organs, particularly the cardiovascular system can also be involved,^2^ leading to serious complications such as pericarditis, myocarditis, acute coronary syndromes, Takotsubo cardiomyopathy, heart failure, arrhythmias, intracardiac or extracardiac thrombus, pulmonary embolism and stroke.^1^,^3^,^4^ Fulminant myocarditis leading to cardiogenic shock and death has also been reported.^5^,^6^ While myocarditis has been reported to occur after SARS-CoV-2 vaccination, the risk is much higher after SARS-CoV-2 infection.^7^ The pathophysiology of COVID-19-related myocarditis is complex and multifactorial, and often believed to be a combination of direct viral injury and cardiac damage due to the host’s immune response.^8^ Nevertheless, international guidelines on the management of COVID-19-related myocarditis are yet to be established,^9^ and most of the evidence comes from published case reports. In the present article, we present the case of a male patient in his early fifties who was admitted with life-threatening myocarditis as a complication to COVID-19 infection. He was successfully treated and had complete recovery without any sequelae.

## CASE PRESENTATION

### Clinical presentation and management

A previously healthy man in his early fifties was admitted to a local hospital with nausea and epigastric pain, but without any symptoms from the respiratory system. He had a positive COVID-19 test two days earlier but had not noted any respiratory symptoms. He had received a first dose of the BNT162b2 mRNA COVID-19 vaccine (Comirnaty, BioNTech/Pfizer) two months prior to the current illness. Three years earlier, he had been investigated for involuntary weight loss, but no cause had been ascertained. He reported moderate alcohol use and smoked 20-30 cigarettes per week.

**Fig.1:**
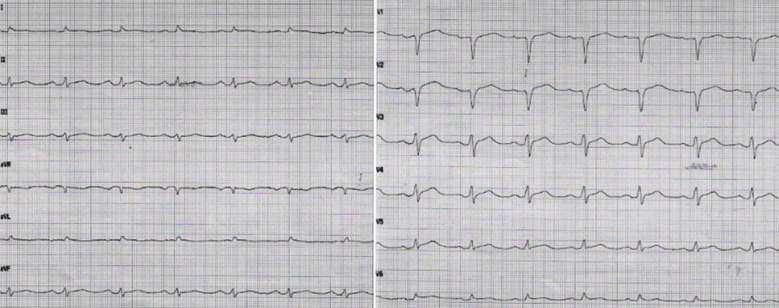
Electrocardiogram at admission showing ST segment elevations in the anterior precordial leads and mild PR segment depression in inferolateral leads.

**Fig.2:**
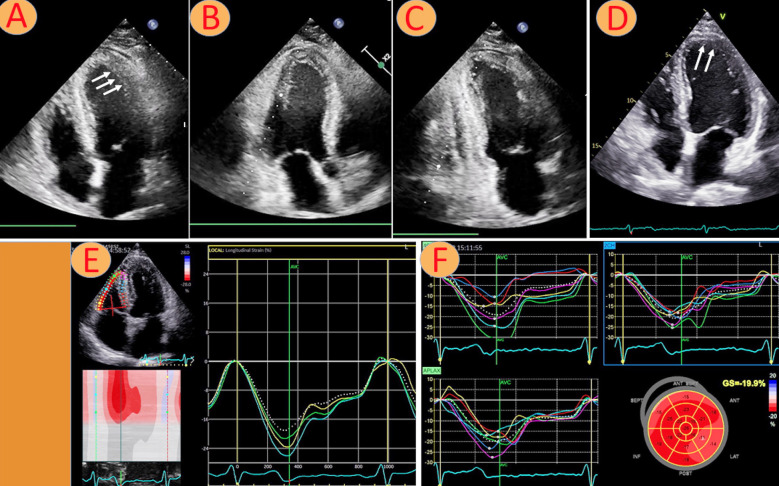
Echocardiographic findings of edema of the Left ventricle (A,B arrows). Significant improvement of left ventricular thickness (C, D arrows). Normalization of right and left ventricular global longitudinal strain (GLS) at 2 month follow-up (E-F).

Echocardiography at admission at a local hospital showed global hypokinesia and apparently thickened myocardium. ECG revealed ST segment elevations in the anterior precordial leads (Fig.1). Coronary angiography showed open coronary arteries. The patient was hemodynamically unstable and was transferred to the cardiology department at our tertiary hospital. Here, the echocardiogram confirmed biventricular failure with a left ventricular (LV) ejection fraction (LVEF) of 10%, thickened interventricular septum (1.6 cm) (Fig.2A-B) (LV outflow track velocity time integral of 4.1 cm, yielding a raw stroke volume of 17 ml (data not shown). Laboratory findings showed elevated troponin T at 1320 ng/L and proBNP at 15000 ng/L. Serum creatinine levels were 158 μmol/L and liver-function tests showed alanine aminotransferase (ALT) at 427 u/L (<70) and aspartate aminotransferase (AST) at 318 u/L (<45) (Table-I). At admission, his throat swab was positive on RT-PCR for SARS-CoV-2 of the *Omicron* variant BA.2. He also had positive immunoglobulin G for SARS-CoV-2. Myocarditis as a complication of COVID-19 infection was the most likely diagnosis. The patient was in cardiogenic shock and was intubated and put on veno-arterial Extracorporeal Membrane Oxygenation (VA-ECMO), and simultaneously on an Impella device for offloading of the LV; and he was then transferred to the Intensive Care Unit (ICU).

**Table-I:** Laboratory findings.

Blood tests	Admission to hospital	Discharge
Troponin T (ng/L)	1320	1200
proBNP (ng/L)	15000	4400
CRP (mg/L)	55	13
Creatinine (μmol/L)	158	58
ALT (u/L)	427	87
AST (u/L)	318	108

### Further Investigations

There was no other active infection except for COVID-19. Serology was positive for immunoglobulin G and negative for immunoglobulin M for Cytomegalovirus, parvovirus B19 and herpes simplex virus (HSV) 1, consistent with prior infection. Serology was negative for immunoglobulin G for HSV 2 and pan-HSV immunoglobulin M. He had immunoglobulin G antibodies against Epstein Barr Virus nuclear antigens (EBNA) also consistent with prior infection. PCR from his blood was negative for human parvovirus B19 DNA. PCR on a myocardial biopsy was negative for DNA from herpes simplex virus 1 and 2, cytomegalovirus, Epstein-Barr-virus, human parvovirus B19 and adenovirus, and negative for enterovirus RNA. PCR from throat swab was negative for influenza virus A and B. Blood cultures were negative. Multiplex PCR was negative for a panel of intestinal pathogens, including adenovirus, norovirus 1 and 2, astrovirus, rotavirus, sapovirus, *Giardia* lamblia, *Entamoeba histolytica*, *Cryptosporidium species*, *Yersinia enterocolitica*, *Campylobacter*, *Salmonella* enteropathogenic *E. coli* and *Clostridium difficile* toxin B. Urine culture was negative for *Pseudomonas aeruginosa* ST3875, a recent cause of nosocomial infections in Norwegian hospitals. He had a positive antigen test for Helicobacter pylori from stools. Extended investigation for rheumatological disease was negative. A cardiac magnetic resonance imaging (CMR) was interrupted due to claustrophobia, but the images obtained confirmed generalized thickening of the myocardium compatible with edema. Myocardial biopsy did not reveal Giant Cells or signs of other underlying cardiomyopathies, but some focal fibrosis of uncertain etiology and intracellular edema (data not shown).

### Treatment and clinical outcome

The patient was treated for COVID-19 with remdesivir 200 mg first day following with 100 mg daily for ten days in total, methylprednisolone 1g per day for three days, intravenous immune globulin 1g/kg for three days. He was extubated and weaned from VA-ECMO six days after admission, and the impella device was removed three days following ECMO removal. Further, he received optimal heart failure medications with angiotensin converting enzyme (ACE) inhibitor ramipril 5mg per day spironolactone 25 mg per day and beta blocker - metoprolol 50 mg per day. LVEF gradually improved and normalized to 60% at discharge. Similarly, LV end-diastolic dimension also reduced from 5.2 cm to 4.1 cm.

### Follow-up

Two months after discharge the patient was assessed at Outpatient Clinic. The interventricular septum thickness was reduced to 1.2 cm (Fig.2C-D). LVEF remained stable at 60%, and right ventricular free wall strain was -24% and LV global longitudinal strain (GLS) -20% (Fig.2E-F). CMR revealed a structurally normal myocardium. A 24-hour electrocardiogram did not show any arrhythmias.

The patient continued treatment with ACE inhibitor and low dose beta blocker (metoprolol 25 mg per day), without mineralocorticoids. Currently, a one-year follow-up at his local hospital is scheduled.

## DISCUSSION

We present the clinical course of COVID-19 related fulminant myocarditis in a previously healthy individual, with successful clinical outcome. Recent SARS-CoV-2 vaccination could in principle have been the cause of myocarditis. However, vaccination-induced myocarditis frequently occurs earlier^10^ and a lag of two months from vaccination to evident myocarditis makes the association less likely. Furthermore, myocarditis is much more likely to occur after SARS-CoV-2 infection than vaccination.^7^

Myocarditis and myocarditis-like injury have been increasingly reported in the literature as complications of COVID-19.^11^ However, the prevalence of COVID-19 related myocarditis is unclear.^12^ The incidence of myocarditis after COVID-19 is estimated to 4 pr 100 000,^7^ increasing to 2-4 per 1000 among hospitalized patients,^13^ and 2%– 30% in autopsy studies of patients who died due to COVID-19.^14^-^16^

The exact mechanism of COVID-19-induced myocarditis is not fully understood, and is complex and multifactorial. However, direct myocardial injury by the coronavirus (virus-induced toxicity) and dysregulated immune response, cytokine-mediated organ damage, ischemia, endothelial injury, edema, inflammation in the arterial wall, subintimal bleeding, coagulopathy and microthrombus have been suggested as possible pathophysiological mechanisms.^17^

Regarding the mechanism of COVID-19-induced myocarditis in the early phase of the pandemic, Hoffman *et al.*, provided useful insights into the first step of SARS-CoV-2 infection and viral entry into cells defining potential targets for antiviral intervention.^9^,^18^

The diagnosis of COVID-19 related myocarditis is based on the clinical presentation, results of blood tests, myocardial biopsy, electrocardiogram and imaging modalities, i.e. echocardiography and CMR. Elevation of both troponin and proBNP levels are observed, combined with electrocardiogram abnormalities such as ST elevation and new onset bundle branch block.^19^,^20^ According to recommendations issued by the American heart association and European Society of Cardiology, patients presenting with symptoms of myocarditis should be further investigated with at least one imaging method such as echocardiogram and/or CMR.^21^,^22^ Echocardiography in individuals with COVID-19 infection showed non-specific patterns of ventricular dysfunction, although new myocardial infarction, myocarditis, and Takotsubo cardiomyopathy were observed in a minority of patients.^23^

In our patient, echocardiography at admission showed significant ventricular dysfunction with generalized wall thickening. Myocardial inflammation and edema which can persist for weeks or even months following initial presentation of COVID-19 infection, have been reported in CMR studies.^24^ CMR in our patient demonstrated thickened myocardium – resembling hypertrophy, which indicated myocardial edema.

Furthermore, myocardial biopsy is recommended for a definite diagnosis of myocarditis with reported histopathologic findings revealing intracellular edema as enlarged cardiomyocytes without interstitial or replacement fibrosis.^21^,^22^,^24^,^25^ Other investigations, such as coronary angiography may be indicated to exclude differential diagnosis of acute coronary syndromes. The patient presented with symptoms of cardiogenic shock and significant elevation of troponin and proBNP. Acute coronary syndrome was excluded with a coronary angiography, and endomyocardial biopsy revealed intracellular edema and no sign of giant cells, considerably supporting the diagnosis of COVID myocarditis.

Management of COVID-19 myocarditis depends on the severity at clinical presentation. Heart failure and/or cardiogenic shock should be treated according to current international guidelines and protocols. For hemodynamically unstable patients, mechanical circulatory support can be life-saving, as in our case.^21^,^22^ While guidelines are lacking, in the presence of signs of active infection, treatment with antivirals such as remdesivir or nirmatrelvir/ritonavir, as well as steroids or intravenous immune globulin, tocilizumab or sarilumab could be considered.^21^

## CONCLUSION

The outcome of COVID-19 related serious myocarditis varies from full recovery to death in some rare high-risk patients. Risk factors for COVID myocarditis are still subject of ongoing research. Our patient was previously healthy, an important factor predicting favorable outcome despite an urgent and unstable initial presentation. Management was according to expert consensus, guidelines for myocarditis and previous experience documented in published literature. The patient was successfully treated and recovered without any sequelae. The pathophysiology and management, as well as individual susceptibility to the disease are not fully understood yet and should be investigated in larger collaborative studies on COVID-19 patients with cardiac involvement.

### Authors’ Contribution:

**VK** did data collection, conceived, designed and editing of manuscript.

**TL, AS, BB** treated the patient, did literature search and edited the manuscript.

**SS** did data collection, treated the patient, conceived, designed and edited the manuscript, and is responsible for integrity of research.

All authors approved the manuscript for submission.
